# The Prevalence of Depression and Depressive Symptoms among Eye Disease Patients: A Systematic Review and Meta-analysis

**DOI:** 10.1038/srep46453

**Published:** 2017-04-12

**Authors:** Yajing Zheng, Xiaohang Wu, Xiaoming Lin, Haotian Lin

**Affiliations:** 1 State Key Laboratory of Ophthalmology, Zhongshan Ophthalmic Center, Sun Yat-sen University, Guangzhou, Guangdong, 510060, People’s Republic of China

## Abstract

The prevalence of depression among different eye disease patients varies across studies and has not been systematically reviewed. This study is to provide a summary of the prevalence of depression among eye disease patients. PubMed, Medline, Embase and Cochrane Library were searched from January, 1990 to December, 2015 to identify studies with information on the prevalence of depression among ophthalmic patients. A random/fixed-effects meta-analysis was used to estimate the pooled prevalence of depression among eye disease patients. Heterogeneity was assessed with the I^2^ test. 28 studies were selected from 3162 references. The overall pooled prevalence of depression or depressive symptoms with eye disease was 25% (1502/6589 individuals, 95% CI, 0.20–0.30) ranging from 5.4% to 57.0%. Regarding different disease categories, the highest prevalence was revealed for dry eye disease (DED) with 29%, followed by 25% for glaucoma patients, 24% for age-related macular degeneration (AMD) patients, 23% for cataract patients. The increased pooled prevalence of depression was identified in those with eye diseases compared with healthy controls (OR, 1.59; 95% CI, 1.40–1.81; I^2^ = 68.5%). Substantial heterogeneity was identified across most estimates (I^2^ > 75%). Further research is needed to identify effective strategies for preventing and treating depression among eye disease patients.

With the transformation from the biomedical to socio-psycho-biological medical model, an increasing attention has been paid on mental health and life quality during disease managements^1^. According to the epidemiological data from World Health Organization (WHO), the prevalence of depression occupies the fourth leading contributor to the global disease burden^2^ and is expected to increase^3^. Depression or depressive symptoms have been proved to be associated with various systemic diseases, including obesity^4^, metabolic disorders^5^ and cardiovascular diseases^6^, contributing to economic burden and social disturbance^3^.

Many studies have paid attention to psychological problems and suggest that mood disorders, particularly depression, are associated with eye disease^7^,^8^,^9^. The progressive vision loss caused by age-related macular degeneration (AMD)^7^, cataract^10^ or glaucoma^11^ constitutes a major source of disability; the chronic ocular discomforts induced by dry eye disease (DED)^12^ or inflammatory disorders^8^ may be related to the deteriorating mode status as well. Timely and effectively detection of the psychological changes will contribute to management of ocular symptoms, improvement of life quality, and also, maintaining peaceful communications between physicians and patients.

However, to our best knowledge, the prevalence of depression among various ophthalmic patients varies across studies^7^,^8^,^10^,^11^,^12^,^13^,^14^,^15^,^16^,^17^,^18^,^19^,^20^,^21^,^22^,^23^,^24^,^25^,^26^,^27^,^28^,^29^,^30^,^31^,^32^,^33^,^34^,^35^ and has not been systemically reviewed. The lowest reported prevalence of depression among eye disease patients is 5.4%^14^, while the highest is 57%^26^. Therefore, a systematic review and meta-analysis was performed to estimate the prevalence of depression among different eye disease patients.

## Results

### Study characteristics

**Figure 1:**
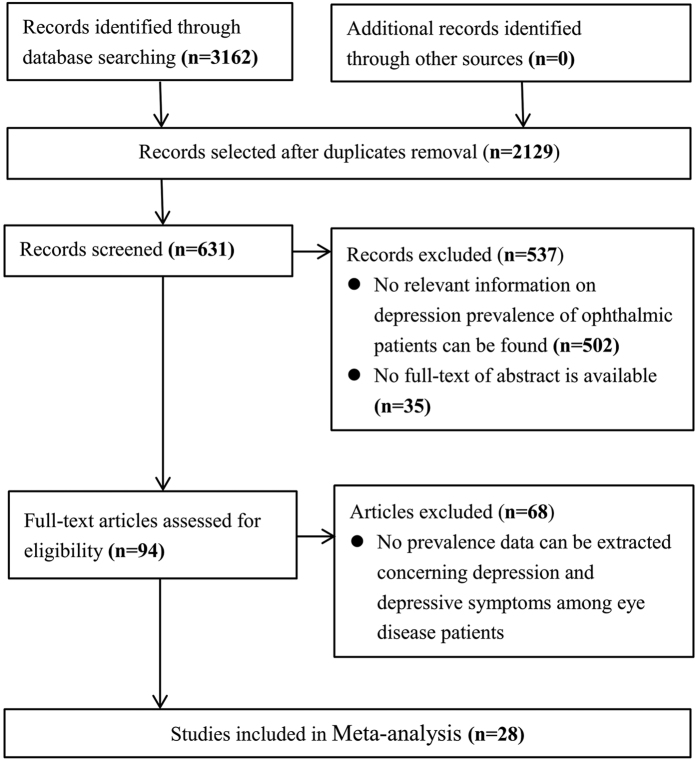
Flowchart of study selection process.

Twenty-eight studies^7^,^8^,^10^,^11^,^12^,^13^,^14^,^15^,^16^,^17^,^18^,^19^,^20^,^21^,^22^,^23^,^24^,^25^,^26^,^27^,^28^,^29^,^30^,^31^,^32^,^33^,^34^,^35^ were finally included in the quantitative synthesis from 3162 original records (Fig. 1). Ten took place in America, 8 in Asia, 7 in Europe (including 1 multicenter study performed across France, Germany and Italy) and 3 in Australia. Eight studies focused on AMD, 5 on DED, 3 on cataract, 12 on glaucoma, and 4 on other eye diseases. Six studies assessed depression using the Hospital Anxiety and Depression Scale (HADS)^36^,^37^, 3 used the Center for Epidemiologic Studies Depression Scale (CES-D)^38^, 6 used the Geriatric Depression Scale (GDS)^39^,^40^, 4 used the Beck Depression Inventory (BDI)^41^, 1 used the 9-items Patients Health Questionnaire (PHQ-9)^42^,^43^, 1 used the Goldberg Anxiety and Depression Scale (GADS)^44^, 2 used the Zung Self-rating Depression Scale (SDS)^45^,^46^, 1 reported no questionnaire, and 4 used other assessed instruments^47^. Regarding quality assessment, 67.9% (19/28) included studies received 3 points of higher, indicating low risk of bias. The prevalence of depression or depressive symptoms among eye disease patients ranged from 5.4%^14^ to 57.0%^11^,^26^ in the systematic review and meta-analysis (Table 1).

### Prevalence of depression among eye disease patients

**Figure 2:**
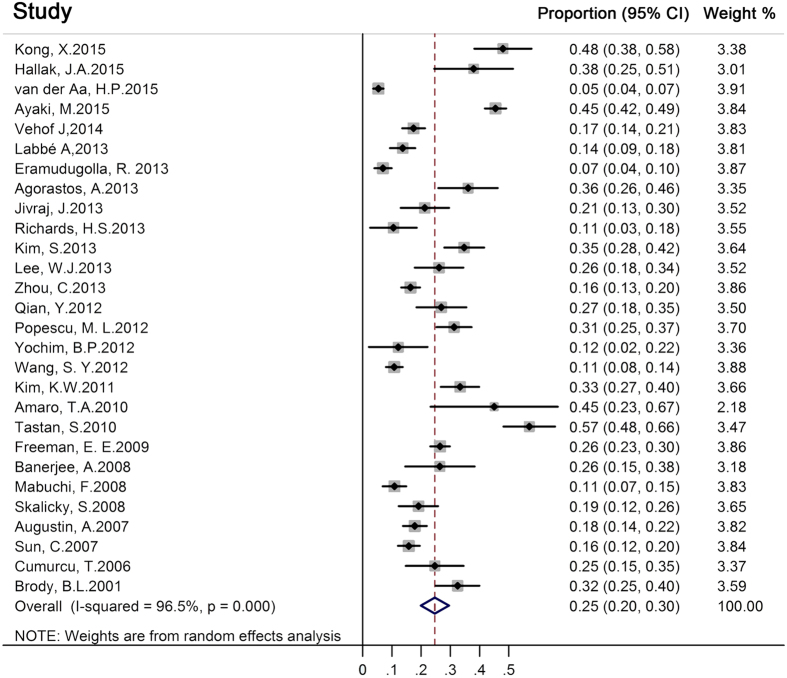
Forest plot of the 28 studies estimating the pooled prevalence of depression among eye disease patients.

**Figure 3:**
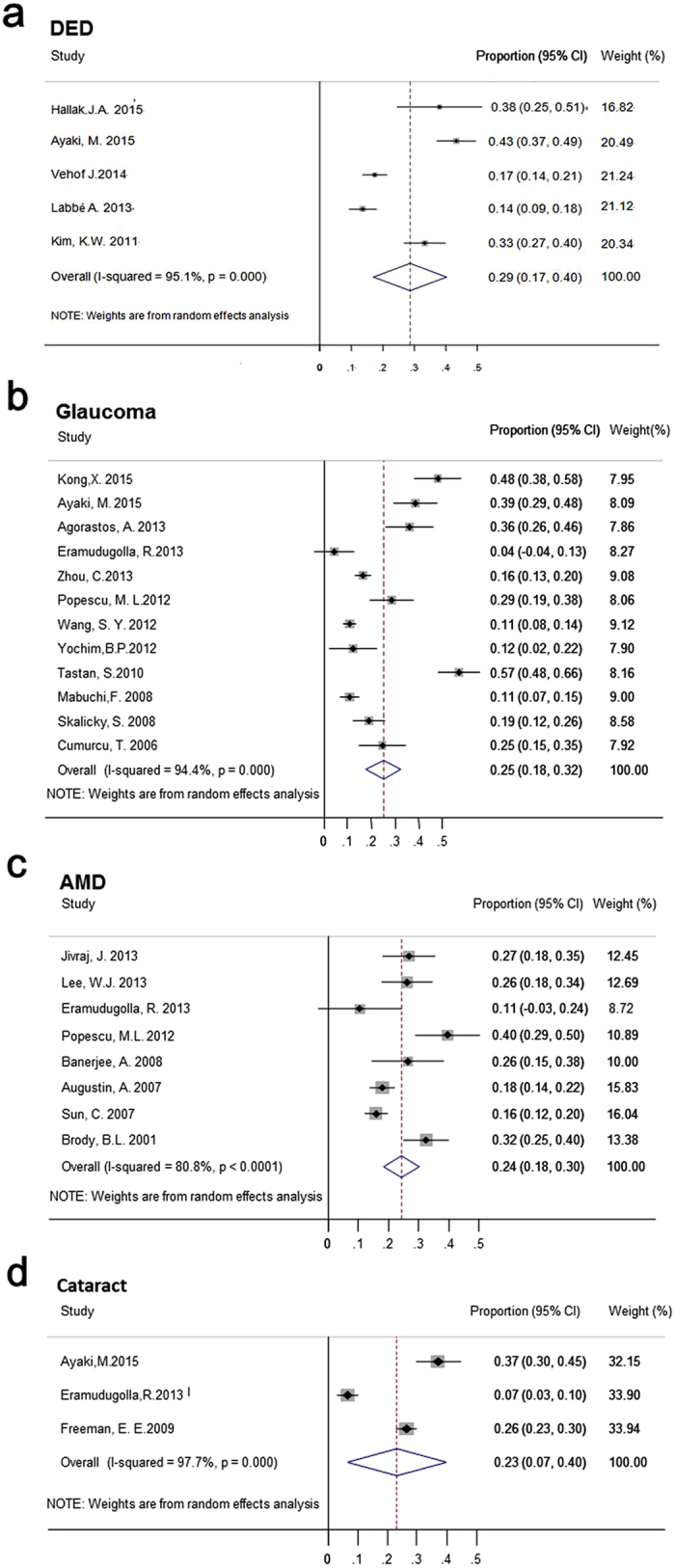
Forest plot of the pooled prevalence of depression in different eye disease patients. (**a**) Forest plot of the pooled prevalence of depression in Dry Eye Disease (DED) patients. (**b**) Forest plot of the pooled prevalence of depression in glaucoma patients. (**c**) Forest plot of the pooled prevalence of depression in Age-related Macular Degeneration (AMD) patients. (**d**) Forest plot of the pooled prevalence of depression in cataract patients.

The overall pooled prevalence of depression or depressive symptoms with eye diseases was 25% (1502/6589 individuals, 95% CI, 0.20–0.30) (Fig. 2). Regarding different eye disease subgroups, the highest depression prevalence was revealed for DED patients 29% (95% CI, 0.17–0.40), followed by 25% (95% CI, 0.18–0.32) for glaucoma patients, 24% (95% CI, 0.18–0.30) for AMD patients, 23% (95% CI, 0.07–0.40) for cataract patients, see Fig. 3. Substantial heterogeneity was identified across most estimates (I^2^ > 75%) (Fig. 3).

### Secondary analysis of 1^2^ studies with control groups

**Figure 4:**
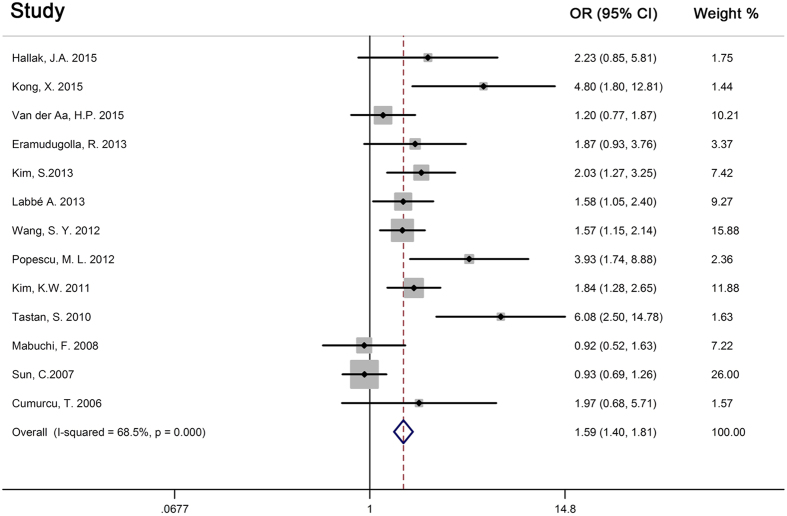
Forest plot of the 13 studies with control groups in meta-analysis.

The 13 of the 28 studies with control groups were included in the meta-analysis. The overall number of eye disease patients with depression or depressive symptoms was 574 (total of 3149), while it was 1092 (total of 12152) in the control group. The odds ratio of the meta-analysis depending on the random-effects model was 1.59 (95% CI 1.40–1.81, Q = 38.11, I^2^ = 68.5%). The forest plot revealed that the prevalence of depression of the control group was lower than that for the group with eye disease, and this difference was significant difference (Fig. 4). The funnel plot of the 13 studies with control groups was generally symmetrical (Supplementary Figure S1). The results of the Begg’s test (*p* = 0.059) revealed no significant publication bias.

## Discussion

This systematic review and meta-analysis demonstrated that the prevalence of depression among eye disease patients was 25% (1502/6589 individuals, 95% CI, 0.20–0.30), ranging from 5.4% to 57%. Regarding different disease categories, the highest prevalence was revealed for DED with 29%, followed by 25% for glaucoma patients, 24% for AMD patients, 23% for cataract patients. More than two thirds of included studies were assessed with low risk of bias, whereas, substantial heterogeneity was identified across most estimates. Depression or depressive symptoms in eye patients may be caused by a series of eye symptoms, which have a great impact on patients’ daily lives, such as eye discomfort, foreign body sensation, pain, visual impairment and other symptoms^10^,^35^. This result may also be caused by social factors, doctor visits and medical expenses. The depressive symptoms of eye disease patients deserve more attention^48^,^49^.

Depressive symptoms may not only aggravate symptoms of eye disease but also affect other psychological systems^50^. For example, these patients usually felt sad, pessimistic and experienced cognitive disorders and pain, forming a vicious circle. With a change of the medical model, people are placing an increasing emphasis on the mental health of patients. It is imperative that social measures be introduced to improve health. These findings highlight an important social health issue in eye disease patients. In recent years, there have been numerous cases of violence against healthcare workers. The identification of patient mental health, including depression symptom is even more important in maintaining peaceful communications between physicians and patients. We hope the results of this study will further help develop effective strategies for preventing and treating depression among eye disease patients, meanwhile, contributing to establishing the harmonious health environment.

This study found that the prevalence of depression among eye patients was higher than that of the control group (OR = 1.59, 95% CI 1.40–1.81). Regarding different eye disease subgroups, the highest depression prevalence was revealed for DED patients 29%, followed by glaucoma, AMD and cataract patients. That indicated the prevalence of depression and depressive symptoms in DED patients was higher than that of other eye disease patients. The underlying theories may involve chronic painful symptoms of dry eye that can cause depression, the medications used to treat depression can cause or exacerbate dry eye, or the same underlying mechanism such as hormonal, metabolic or neurological imbalance that results in depression can also cause dry eye disease. Additionally, depression might affect the management and outcomes of dry eye treatment. Some reports also described DED as being closely associated with depression^10^,^12^. Aged patients are especially sensitive to negative feelings of helplessness. Some studies demonstrated that relieving eye discomfort symptoms and promoting visual rehabilitation have a positive impact on depression of ophthalmic patients^51^,^52^. More efforts are needed to identify relevant factors inducing depression and depressive symptoms among eye disease patients and to provide appropriate prevention and treatment for mental disorders. Effective treatment of depression and screening tools are also necessary. Ophthalmologists should cooperate with psychiatrists to treat eye disease and improve mental health.

This review has a few limitations. **First**, the sample sizes of the included studies contributed to the observed heterogeneity in the data. Studies with small sample size might have yielded more extreme prevalence estimates, suggesting the presence of publication bias. **Second**, the diagnosis for depression or depressive symptoms was not uniform between studies with diverse screening instruments and cutoff scores. Additionally, the diagnosis for the eye diseases was even more complicated, contributing partially to the heterogeneity. **Third**, most studies involve cross-sectional or retrospective data. Also, individuals with chronic eye diseases are more likely to be screened for depression symptoms than the subjects without. **Fourth**, most of the included participants were aged patients and would probably suffer from systemic diseases, which may induce depression. That could be a potential confounding factor for the estimation. Therefore, a multicenter prospective study using standard diagnostic methods and screening tools on a random subset of participants would confirm this association and provide a more accurate estimate of the prevalence of depression among eye disease patients. Despite the above limitations, the findings of this study suggest that the improvement of treatment of eye disease may reduce the prevalence of depression or depressive symptoms. Conversely, it might suggest that appropriate management of eye disease associated depression symptoms may improve the effectiveness of eye disease treatment and the quality of life of these patients.

In conclusion, the overall prevalence of depression among eye disease patients was 25%, varying with disease categories. The prevalence of depression among eye disease patients was higher than that in healthy people. Further research is needed to identify effective strategies for preventing and treating depression among eye disease patients.

## Methods

### Criteria for including studies and searching methods

Descriptive studies and cross-sectional studies that reported depression or depressive symptoms among people with eye diseases were included in the systematic review. Studies were included if they were published in peer-reviewed journals, and the subjects were assessed of depression or depressive symptoms using a validated method. The diagnosis of eye diseases were based on the judgment of qualified ophthalmologists or on medical records showing a diagnosis according to the International Classification of Disease and Codes (ICD-11). The electronic databases PubMed, Medline, Embase and Cochrane Library were searched for the relevant studies published from January, 1990 to December, 2015 following the instructions of the Preferred Reporting Items for Systematic Review and Meta-Analyses (PRISMA) guidelines (Fig. 1)^53^,^54^. We also manually checked the reference lists of all retrieved studies. In our literature search, we included a combination of keywords, such as depression/depressive symptoms, prevalence/incidence, eye disease/ophthalmology, etc, in the form of title words or medical subject headings. For details, please refer to Supplementary Appendix A. Two reviewers (Y.Z. and X.W.) completed the literature search independently. In addition, these two reviewers further cross-checked the reference lists of all selected articles to identify other relevant studies. When screening discrepancies occurred, consensus was achieved after further discussion.

### Data extraction and quality assessment

Data extraction from each study used a standardized form: study design, geographic location, sample size, average age of participants, disease category, diagnostic or screening method used outcome definition (cut off for specific diagnostic criteria or screening instrument), and reported prevalence of depression or depressive symptoms^55^. The Newcastle-Ottawa Scale (NOS) was used to assess the quality of non-randomized studies included in the systematic review and meta-analysis^56^, recommended by the Agency for Healthcare Research and Quality (AHRQ), available at http://www.ohri.ca/programs/clinical_epidemiology/oxford.asp. This scale uses a star system to assess the quality of a study in three domains: selection of study groups; comparability of groups; and ascertainment of outcomes. Studies were judged to be at low risk of bias (3 points or more) or high risk of bias (<3 points).

### Data synthesis and analysis

Prevalence estimates of depression or depressive symptoms were calculated by meta-analysis based on a proportions approach for the included 28 studies and for different eye disease subgroups. Between-study heterogeneity was assessed using standard x^2^ tests and the I^2^ statistic^57^,^58^. I^2^ values of 50% or more were considered to indicate substantial heterogeneity, and the random-effects model was then used; otherwise, the fixed-effects model was used^59^. Thirteen studies with a control group were included in the secondary meta-analysis used the I^2^ statistic and the random-effect model to analyze whether there was a statistically significant difference between the subgroups with eye disease and the healthy people. A funnel plot and the Begg’s test were used to investigate the publication bias between the twelve studies^60^,^61^. All analyses were performed using Stata (version 12.0, Stata Corp., College Station, TX) and R (version 3.2.1, The R Foundation, Vienna, Austria). Statistical tests were 2-sided and used a significance threshold of *p* < 0.05.

## Additional Information

**How to cite this article**: Zheng, Y. *et al*. The Prevalence of Depression and Depressive Symptoms among Eye Disease Patients: A Systematic Review and Meta-analysis. *Sci. Rep.* **7**, 46453; doi: 10.1038/srep46453 (2017).

**Publisher's note:** Springer Nature remains neutral with regard to jurisdictional claims in published maps and institutional affiliations.

## Supplementary Material

Supplementary Information

## Figures and Tables

**Table 1:** Characteristics of 28 included studies in the systematic review and meta-analysis.

Study	Country	Disease	Age, y	Study Design	Diagnostic Method	Outcome	Depression Prevalence (case/participant)	NOS
Kong, X.^11^	China	Glaucoma	Mean (SD) 55.5 (NR)	Cross-sectional study	SDS	≥50	48% (48/100)	2
Hallak, J. A.^13^	US	DED	Mean (SD) 52.6 (16.2)	Cross-sectional study	BDI	>9	38% (19/50)	3
Van der Aa, H. P.^14^	Netherland	Eye disease	Mean (SD) 77.6 (9.3)	Cross-sectional study	CES-D	≥16	5% (33/615)	3
Ayaki, M.^10^	Japan	Eye disease	Mean (SD) 59.5 (19.9)	Descriptive study	HADS	≥10	45% (332/730)	3
Vehof J.^34^	British	DED	Mean (SD) 57.1 (13.1)	Cross-sectional study (population-based)	Clinical diagnosis	NR	17% (64/367)	3
Labbé A^35^	China	DED	Mean (SD) 67.3 (9.6)	Cross-sectional study (population-based)	SDS	≥40	14% (33/241)	3
Eramudugolla, R.^15^	Australia	Age-related eye disease	Mean (range) NR (>70)	Cross-sectional study (population-based)	GADS	NR	7% (20/287)	1
Agorastos, A.^16^	Germany	Glaucoma	Mean (range) NR (>50)	Descriptive study	BDI-II	≥10	36% (31/86)	3
Jivraj, J.^17^	Canada	AMD	Mean (range) NR (>50)	Descriptive study	CES-D	≥16	21% (20/94)	4
Richards, H. S.^18^	UK	Ptosis	Mean (SD) 61.6 (15.3)	Descriptive study	HADS	>10	11% (6/57)	2
Kim, S.^19^	Korea	RP	Mean (SD) 40.1 (11.0)	Cross-sectional study (population-based)	NR	NR	35% (65/187)	1
Lee, W. J.^20^	Korea	AMD	Mean (SD) 55 (NR)	Descriptive study	GDS	≥5	26% (28/107)	3
Zhou, C.^21^	China	Glaucoma	Mean (SD) 55.4 (15.3)	Descriptive study	HADS	>10	16% (83/506)	3
Qian, Y.^8^	US	Ocular inflammation	Mean (range) NR (>18)	Descriptive study	BDI	>13	27% (28/104)	2
Popescu, M. L.^22^	Canada	Eye disease	Mean (range) NR (>65)	Cross-sectional study	GDS-15	≥5	31% (71/227)	4
Yochim, B. P.^23^	US	Glaucoma	Mean (SD) 70 (9.2)	Descriptive study	GDS	≥5	12% (5/41)	3
Wang, S. Y.^24^	US	Glaucoma	Mean (SD) 66.9 (1.03)	Cross-sectional study (population-based)	PHQ-9	≥10	11% (49/453)	4
Kim, K. W.^12^	Korea	DED	Mean (SD) 71. 9 (5.8)	Cross-sectional study (population-based)	SGDS-K	NR	33% (66/198)	1
Amaro, T. A.^25^	US	Uveal melanoma	Mean (range) 52 (29–80)	Descriptive study	BDI	>11	45% (9/20)	3
Tastan, S.^26^	Turkey	Glaucoma	Mean (SD) 64.2 (13.2)	Cross-sectional study	HADS	≥8	57% (69/121)	4
Freeman, E. E.^27^	Canada	Cataract	Mean (range) NR (>45)	Descriptive study	GDS-30	≥10	26% (174/657)	2
Banerjee, A.^28^	US	AMD	Mean (range) NR (>50)	Descriptive study	GDS	>4	26% (14/53)	3
Mabuchi, F.^29^	Japan	Glaucoma	Mean (SD) 66.9 (12.1)	Cross-sectional study	HADS	>10	11% (25/230)	3
Skalicky, S.^30^	Australia	Glaucoma	Mean (range) NR (70–90)	Descriptive study	GDS-15	>4	19% (25/131)	3
Augustin, A.^7^	France Germany Italy	AMD	Mean (SD) 77 (NR)	Descriptive study	HADS	≥11	18% (60/336)	4
Sun, C.^31^	Australia	AMD	Mean (range) NR (69–97)	Cross-sectional study	CES-D	≥10	16% (58/367)	3
Cumurcu, T.^32^	Turkey	Glaucoma	Mean (range) NR (30–80)	Cross-sectional study	HDRS	NR	25% (18/73)	4
Brody, B. L.^33^	US	AMD	Mean (SD) 80 (NR)	Descriptive study	DSM	DSM-IV criteria	32% (49/151)	1
Total	\	\	\		\	\	25% (1502/6589)	\

NR = Not related; Abbreviations: AMD, Age-related Macular Degeneration; DED, Dry Eye Disease; RP, Retinitis Pigmentosa; SDS, Self-Rating Depression Scale; HADS, Hospital Anxiety and Depression Scale; BDI, Beck Depression Inventory; CES-D, Center for Epidemiologic Studies Depression Scale; DSM, Diagnostic and Statistical Manual of Mental Disorders (Forth Edition);HADS, Hospital Anxiety and Depression Scale; NOS, Newcastle-Ottawa Score; NR, not reported; PHQ-9,9-item Patient Health Questionnaire; GDS, Geriatric Depression Scale; SGDS-K, the Korean version of Short Geriatric Depression Scale; SDS, Self-rating Depression Scale; GADS, Goldberg Anxiety and Depression Scale; HDRS, Hamilton Depression Rating Scale.
