# Supplementary material for: The Prevalence of Depression and Depressive Symptoms among Eye Disease Patients: A Systematic Review and Meta-analysis
Source: Sci Rep. 2017 Apr 12;7:46453. doi: 10.1038/srep46453 (PMC5388862; doi:10.1038/srep46453)
Supplement: Supplementary Information [file srep46453-s1.pdf]

Supplementary information

Title Page

The Prevalence of Depression and Depressive Symptoms among Eye Disease Patients: A Systematic Review and Meta-analysis

Yajing Zheng[*], Xiaohang Wu[*], Xiaoming Lin[§], Haotian Lin[§]

Institution: State Key Laboratory of Ophthalmology, Zhongshan Ophthalmic Center, Sun Yat-sen University, Guangzhou, Guangdong, 510060, People's Republic of China

[*]These authors contributed equally to this work and should be considered co-first authors.

[§]**Correspondence:** Haotian Lin, M.D., Ph.D., E-mail: haot.lin@hotmail.com; Xiaoming Lin, M.D., Ph.D., E-mail: linxiaom@mail.sysu.edu.cn; Address: Zhongshan Ophthalmic Center, Xian Lie South Road 54#, Guangzhou, China, 510060. Telephone number: +86-020-87330493, Fax: +86-020-87333271.

Supplementary. Figure S1.

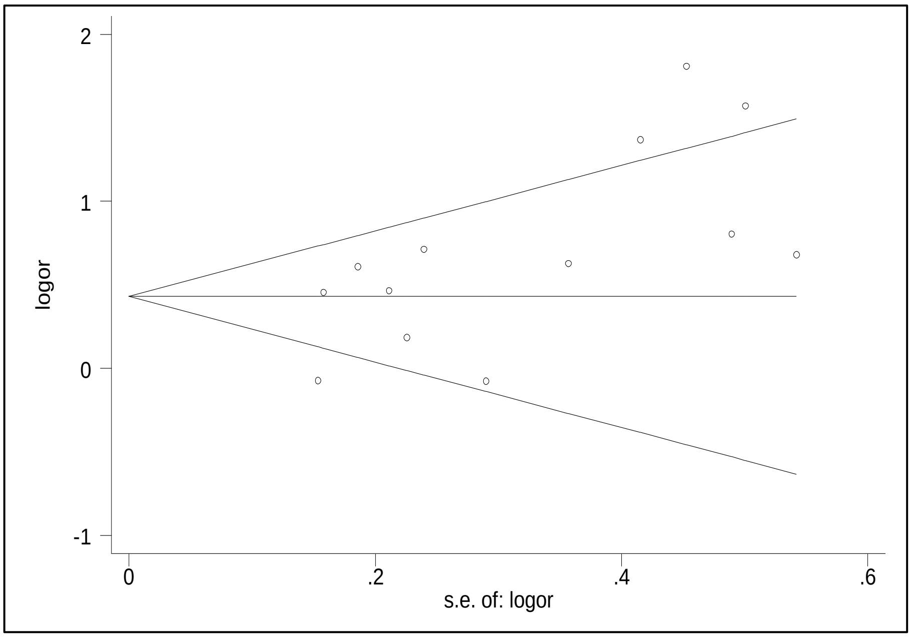

Supplementary. Figure S1. Funnel plots for publication bias testing of the 13 studies with control groups in meta-analysis. Each point represents a separate study on the indicated association. The horizonal line represents the mean effect size. The points are distributed asymmetrically, indicating the existence of publication bias.

Supplementary. Appendix A. PubMed Search Strategy

1. ((Depression[Title/Abstract]) OR Depressive symptoms[Title/Abstract])

2. (((((((Eye disease[Title/Abstract]) OR Ophthalmology[Title/Abstract]) OR Ocular disorder[Title/Abstract]) OR Dry eye disease[Title/Abstract]) OR Glaucoma[Title/Abstract]) OR Age-related macular degeneration[Title/Abstract]) OR Cataract[Title/Abstract])

3. ((((Incidence[Title/Abstract]) OR Prevalence[Title/Abstract]) OR Occurrence[Title/Abstract]) OR Rate[Title/Abstract])

Final PubMed search

(1 AND 2) AND (3).
